# Open-label Phase 1 study to evaluate drug-drug interactions between ulonivirine and islatravir in adults without HIV

**DOI:** 10.1128/aac.00676-26

**Published:** 2026-10-08

**Authors:** Jesse C. Nussbaum, Michelle M. Pham, Ryan Vargo, Yang Liu, Ilias Triantafyllou, Natasha Boyette, Randolph P. Matthews, Marian Iwamoto, Gillian Gillespie

**Affiliations:** 1 Merck & Co., Inc. 2793 , Rahway, New Jersey, USA; University of Houston, Houston, Texas, USA

**Keywords:** pharmacokinetics, drug-drug interactions, non-nucleoside reverse transcriptase inhibitor, human immunodeficiency virus, clinical study

## Abstract

Ulonivirine is a non-nucleoside reverse transcriptase inhibitor in clinical development for once-weekly oral treatment of HIV-1 in combination with islatravir. This open-label, 3-period, Phase 1 study (MK-8507-016, NCT06619678) in adults without HIV evaluated the potential drug-drug interactions (DDIs) between ulonivirine and islatravir. In Period 1, participants received islatravir 2 mg on Day 1. In Period 2, participants received ulonivirine 200 mg on Days 1 and 8. In Period 3, a single dose of islatravir 2 mg was co-administered with ulonivirine 200 mg 7 days after the Day 8 dose in Period 2, followed by four doses of ulonivirine 200 mg once weekly. Thirty-six participants (58% female, 67% White, median age 43 years) were enrolled. Plasma pharmacokinetics of islatravir and ulonivirine were similar with and without coadministration. The 90% *CIs* for geometric mean ratios of co-administration versus single administration of islatravir (AUC_0-168_, 1.06 [1.02–1.09]; C_168_, 1.05 [0.98–1.12]; C_max_, 1.10 [1.02–1.19]) and ulonivirine (AUC_0-168_, 1.07 [1.05–1.09]; C_168_, 1.08 [1.04–1.12]; C_max_, 1.03 [0.98–1.07]) were within standard bioequivalence bounds (0.8–1.25). During Period 3 (co-administration with ulonivirine), intracellular ISL-triphosphate AUC_0-168_, AUC_0-inf_, C_max_, and C_168_ were approximately 21%, 15%, 16%, and 10% lower, respectively, compared with ISL alone. These differences may in part reflect variability in peripheral blood mononuclear cell counts. Adverse events, laboratory test results, and other safety parameters were comparable among all three periods. In conclusion, no clinically meaningful DDIs were observed between ulonivirine 200 mg and islatravir 2 mg, supporting continued development of this combination for HIV-1 treatment.

## INTRODUCTION

HIV continues to pose a major global public health challenge, with an estimated 40.8 million people living with HIV and approximately 1.3 million new infections reported in 2024 (1). Most of the currently available HIV treatment regimens require daily oral dosing, which comes with challenges such as pill fatigue, stigma, and suboptimal adherence (2, 3). A single long-acting complete antiretroviral combination is currently approved for the treatment of HIV-1: cabotegravir with rilpivirine intramuscular injection given once monthly or every 2 months (4, 5). There remains a need for long-acting oral regimens that avoid the logistical challenges of regular injections, injection site reactions, the risk of breakthrough infections, and the emergence of HIV-1 resistance (6). An oral treatment option with reduced dosing frequency could improve lifelong adherence and quality of life (6).

Ulonivirine (ULO, MK-8507) is a non-nucleoside reverse transcriptase inhibitor (NNRTI) in clinical development for weekly oral treatment of HIV-1 in combination with islatravir (ISL), a nucleoside reverse transcriptase translocation inhibitor (NRTTI) (7, 8). ULO is a potent non-competitive inhibitor of reverse transcriptase; similar to other NNRTIs, it binds to an allosteric pocket and induces a conformational change in the substrate-binding site, thereby disrupting reverse transcriptase activity and inhibiting HIV-1 replication (8, 9). ISL is phosphorylated intracellularly to its pharmacologically active islatravir triphosphate metabolite (ISL-TP), which blocks viral replication by inhibition of translocation (immediate chain termination) and induction of structural changes of viral DNA (delayed chain termination) (10–12). Both ISL and ULO demonstrate high activity against wild-type HIV-1 and variants with common resistance-associated mutations (7, 12–16). The long half-lives of ISL-TP and ULO (approximately 195 and 70 h, respectively) suggest that both of these antiviral agents have potential to achieve sustained virologic suppression with weekly administration (7, 17). Dose-dependent decreases in total lymphocyte and CD4+ T-cell counts were observed throughout the ISL development program, which was followed by a trend to recovery after discontinuation (18–21). The observed lymphocyte decreases are believed to be due to intracellular accumulation of ISL-TP, leading to cytotoxicity at higher ISL doses (22).

In a previous drug interaction study, a single 20-mg dose of ISL had minimal impact on the plasma pharmacokinetics (PK) of ULO 400 mg (8). However, a modest increase in plasma ISL was seen, and the PK of ISL-TP were not measured (8). The present study was conducted to more fully characterize the drug interaction between ULO and ISL, including the measurement of ISL-TP in peripheral blood mononuclear cells (PBMCs). The doses of ISL and ULO in the present study were lower than in the previous study, as additional data have informed on the potential therapeutic dose range for weekly administration (22, 23).

## RESULTS

### Study population

A total of 36 participants were enrolled and 35 (97%) completed the study, which was conducted from 17 January to 28 June 2024. The majority of participants were female (58%), White (67%), and not Hispanic or Latino/a (89%); the median age (range) was 43 (19–62) years (Table 1).

**TABLE 1:** Participant demographics and baseline characteristics

Characteristic	Overall *N* = 36
Age, median (range), y	43 (19–62)
Female, *n* (%)	21 (58)
Race, *n* (%)	
White	24 (67)
Black or African American	5 (14)
Multiple^*a*^	5 (14)
Asian	2 (6)
Ethnicity, *n* (%)	
Not Hispanic or Latino/a	32 (89)
BMI, median (range), kg/m^2^	26.7 (22.1–31.6)

^*a*^ Multiple races included White, American Indian or Alaska Native (n = 1); White, Asian (n = 1); and White, Black or African American (n = 3). BMI, body mass index.

Overall, 36 participants were included in the safety and PK analyses for Period 1 (ISL alone), and 35 participants were included in the safety and PK analyses for Period 2 (ULO alone) and Period 3 (ULO plus ISL) (Fig. 1). One participant discontinued the study 52 days after dosing in Period 1 due to multiple adverse events (AEs) that were considered not related to ISL. This participant completed all termination procedures, unscheduled safety assessments, and PK sampling before discontinuation.

**Fig 1:**
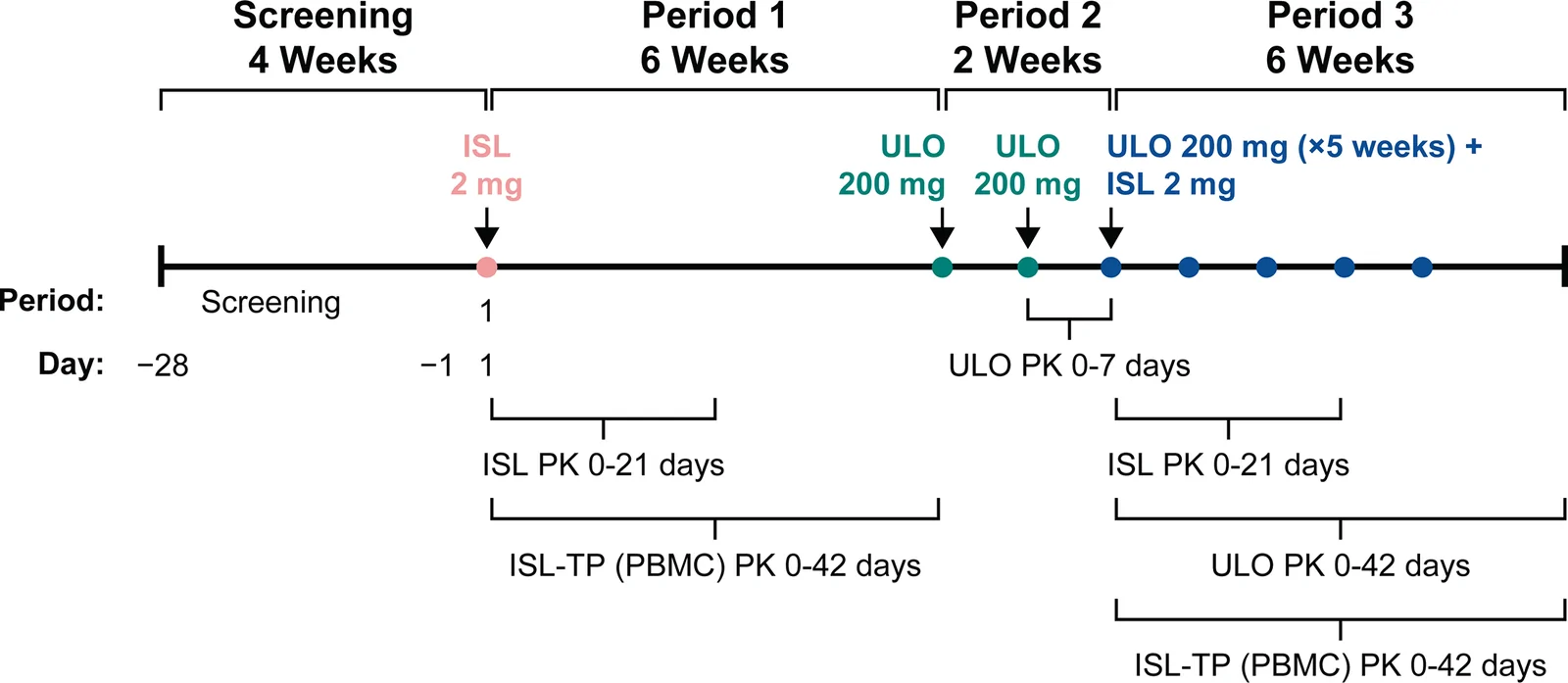
Study design. ISL, islatravir; PBMC, peripheral blood mononuclear cell; PK, pharmacokinetics; TP, triphosphate; ULO, ulonivirine. Period 3 began 7 days (168 h) after Period 2, Day 8.

### PK profile of plasma ISL and intracellular ISL-TP

The plasma ISL and intracellular ISL-TP concentrations were similar with and without co-administration of ULO (Fig. 2). The median time to maximum observed concentration (T_max_) of plasma ISL and intracellular ISL-TP was approximately 0.5 h and 24 h, respectively, following administration of ISL alone and with co-administration of ULO, and apparent terminal half-life (t_1/2_) values were comparable for both plasma ISL and PBMC ISL-TP following both treatments (Table 2).

**Fig 2:**
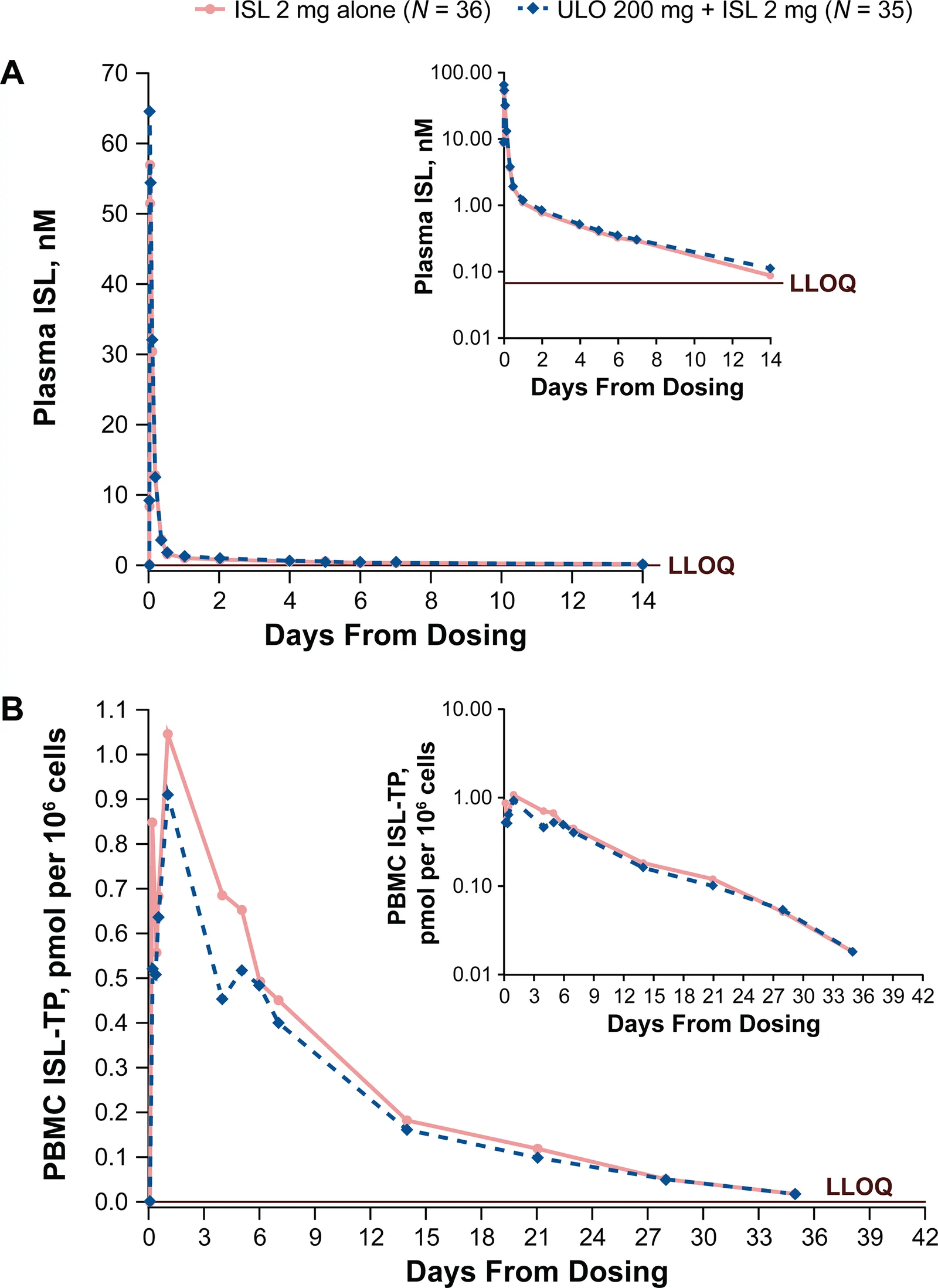
Arithmetic mean plasma concentration-time profiles of (**A**) plasma ISL and (**B**) PBMC ISL-TP following a single dose of ISL 2 mg with and without multiple dosing of ULO 200 mg qw. ISL, islatravir; LLOQ, lower limit of quantitation; PBMC, peripheral blood mononuclear cell; qw, once weekly; TP, triphosphate; ULO, ulonivirine. Main panel: linear scale; inset: semilogarithmic scale. The red reference line refers to the LLOQ = 0.0682 nM.

**TABLE 2:** Summary statistics for plasma ISL and PBMC ISL-TP PK^*a*^

Parameter	ISL *N* = 36	ISL + ULO *N* = 35	(ISL + ULO)/ISL
	Geometric LSM (95% CI)	Geometric LSM (95% CI)	GMR (90% CI)
**ISL**			
AUC_0-168_, h·nmol/L	245 (225–266)	259 (237–283)	1.06 (1.02–1.09)
AUC_0-∞_, h·nmol/L	288 (263–315)	309 (280–341)	1.07 (1.03–1.11)
C_168_, nmol/L	0.273 (0.244–0.306)	0.287 (0.255–0.323)	1.05 (0.98–1.12)
C_max_, nmol/L	58.5 (52.2–65.6)	64.6 (57.7–72.4)	1.10 (1.02–1.19)
T_max_, median (range), h	0.51 (0.49–2.00)	0.50 (0.49–1.02)	NA
t_1/2_, h^*b*^	108 (24.2)	117 (21.1)	NA
CL/F, L/h^*b*^	23.7 (27.1)	22.1 (30.0)	NA
Vd/F, L^*b*^	3,690 (23.8)	3,740 (23.6)	NA
**ISL-TP**			
AUC_0-168_, h·pmol/10^6^ cells	113 (103–125)	89.7 (80.1–100)	0.79 (0.74–0.85)
AUC_0-∞_, h·pmol/10^6^ cells^*c*^	210 (192–229)	178 (160–198)	0.85 (0.80–0.91)
C_168_, pmol/10^6^ cells	0.395 (0.333–0.468)	0.356 (0.303–0.418)	0.90 (0.80–1.01)
C_max_, pmol/10^6^ cells	1.11 (0.993–1.24)	0.929 (0.829–1.04)	0.84 (0.77–0.91)
T_max_, median (range), h	24.04 (4.01–168.04)	24.01 (4.01–143.99)	NA
t_1/2_, h^*b*^	156 (28.2)	176 (26.2)	NA

^*a*^ AUC_0–168_, area under the concentration-time curve from 0 to 168 h after dosing; AUC_0–∞_, area under the concentration-time curve from 0 to infinity; C_168_, concentration at 168 h after dosing; CL/F, apparent clearance after dosing; C_max_, maximum observed concentration; GCV, geometric coefficient of variation; GMR, geometric mean ratio; ISL, islatravir; LSM, least squares mean; NA, not applicable; PBMC, peripheral blood mononuclear cell; PK, pharmacokinetics; t_1/2_, apparent terminal half-life; T_max_, time to maximum drug concentration; TP, triphosphate; ULO, ulonivirine; Vd/F, apparent volume of distribution during the terminal phase after dosing.

^*b*^ Values presented as geometric mean (GCV%).

^*c*^ The number of participants was 33 for the ISL group and 32 for the ISL plus ULO group. Four participants following administration of ISL alone had PBMC ISL-TP AUC_0-∞_ and t_½_ set to missing. Two participants following administration of ULO + ISL had PBMC ISL-TP AUC_0-∞_ and t_½_ set to missing.

The plasma ISL area under the concentration-time curve from 0 to 168 h after dosing (AUC_0–168_), AUC from 0 to infinity (AUC_0–∞_), maximum observed concentration (C_max_), and concentration at 168 h after dosing (C_168_) following co-administration with ULO were approximately 6%, 7%, 10%, and 5% higher, respectively, compared with ISL alone, and the 90% *CIs* for the geometric mean ratios (GMRs; [ISL + ULO]/ISL alone) for these parameters were all confined within standard bioequivalence bounds of 0.8–1.25 (Table 2).

The intracellular ISL-TP AUC_0–168_, AUC_0–∞_, C_max_, and C_168_ after ISL plus ULO co-administration were approximately 21%, 15%, 16%, and 10% lower, respectively, compared with ISL alone (Table 2). These modest decreases in ISL-TP exposure were inversely correlated with differences in PBMC counts (Table S1), which were generally higher after co-administration of ISL plus ULO than with ISL alone across the full range of time points.

### PK profile of plasma ULO

The plasma ULO concentrations were similar with and without co-administration of ISL (Fig. 3). The median T_max_ of plasma ULO was approximately 4 h following both administration of ULO alone and when co-administered with ISL, and the t_1/2_ was comparable after ULO was administered alone and when co-administered with ISL (77.6 versus 80.5 h). The plasma ULO AUC_0–168_, C_168_, and C_max_ following ULO with ISL were approximately 7%, 8%, and 3% higher, respectively, compared with that following ULO alone (Table 3), and the 90% *CIs* of the GMRs ([ISL + ULO]/ULO alone) for these parameters were all confined within the standard bioequivalence bounds of 0.8–1.25 (Table 3).

**Fig 3:**
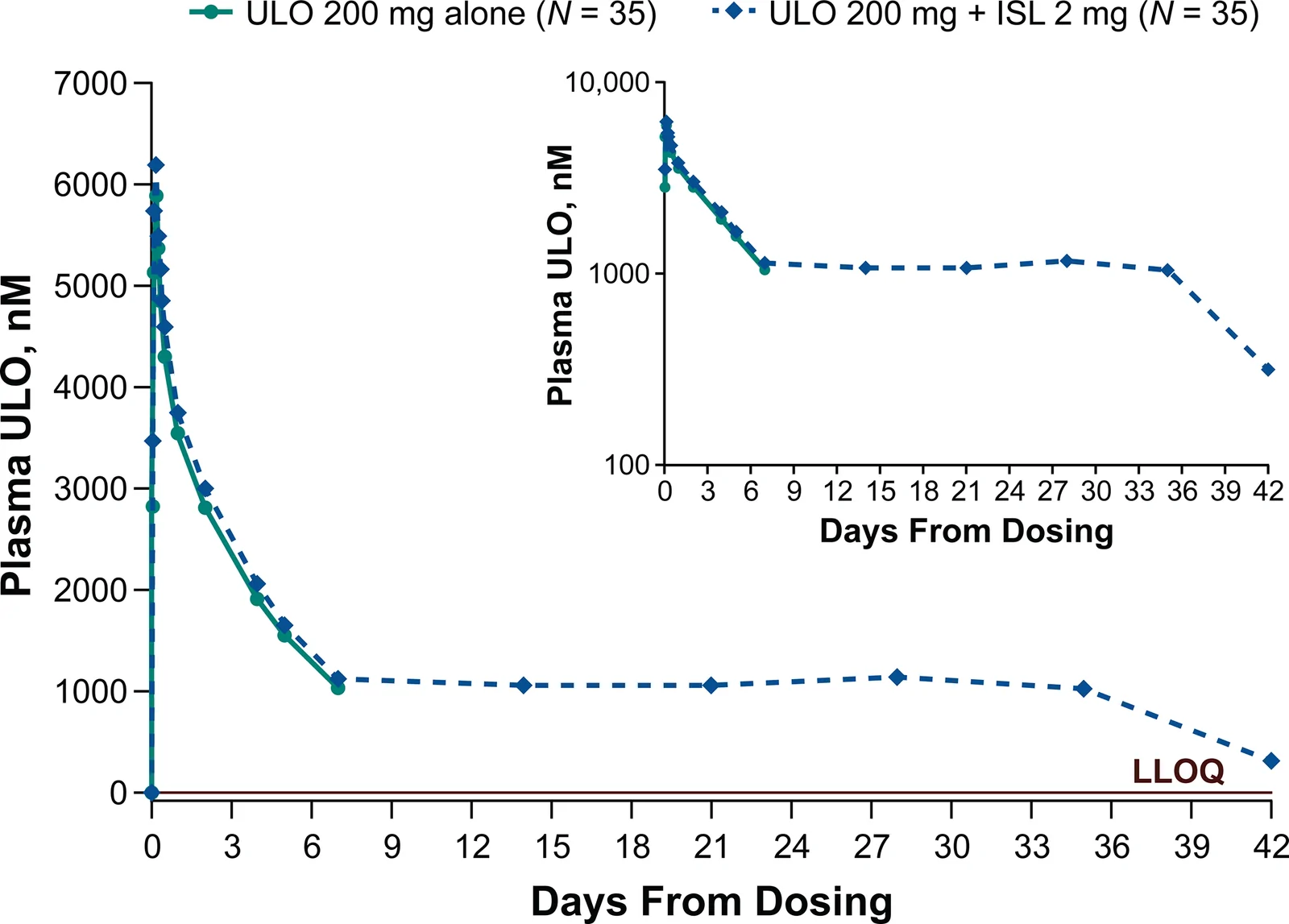
Arithmetic mean plasma concentration-time profiles of ULO after qw dosing with and without a single dose of ISL 2 mg. ISL, islatravir; LLOQ, lower limit of quantitation; qw, once weekly; ULO, ulonivirine. Main panel: linear scale; inset: semilogarithmic scale. The red reference line refers to the LLOQ = 2.03 nM.

**TABLE 3:** Summary statistics for ULO plasma PK^*a*^

	ULO Geometric LSM (95% CI) *N* = 35	ISL + ULO Geometric LSM (95% CI) *N* = 35	(ISL + ULO)/ULO GMR (90% CI)
AUC_0-168_, h·nmol/L	380,000 (345,000–418,000)	407,000 (370,000–447,000)	1.07 (1.05–1.09)
C_168_, nmol/L	961 (830–1,110)	1,030 (889–1,200)	1.08 (1.04–1.12)
C_max_, nmol/L	6,540 (6,010–7,130)	6,710 (6,170–7,310)	1.03 (0.98–1.07)
T_max_, median (range), h	4.00 (2.00–6.00)	4.00 (1.99–8.01)	NA
t_1/2_, h^*b*^	77.6 (19.2)	80.5 (23.4)	NA
CL/F, L/h^*b*^	1.07 (28.8)	1.00 (28.0)	NA
Vd/F, L^*b*^	120 (26.6)	116 (26.9)	NA

^*a*^ AU_0–168_, area under the concentration-time curve from 0 to 168 h after dosing; C_168_, concentration at 168 h; CL/F, apparent clearance after dosing; C_max_, maximum observed concentration; GCV, geometric coefficient of variation; GMR, geometric mean ratio; ISL, islatravir; LSM, least squares mean; NA, not applicable; PK, pharmacokinetics; t_1/2_, apparent terminal half-life; T_max_, time to maximum drug concentration; ULO, ulonivirine; Vd/F of distribution during the terminal phase after dosing.

^*b*^ Values presented as geometric mean (GCV%).

### Safety

A total of 17 participants (47%) reported at least one AE during the study, all of which were mild or moderate. Of the total 81 AEs reported, 23 (28.4%) were considered by the investigator as related to ISL and 37 (45.7%) to ULO. No trends in drug-related AEs were observed, with similar proportions of participants reporting drug-related AEs across groups: 22% (8/36) following ISL alone, 23% (8/35) after ULO alone, and 26% (9/35) after ISL plus ULO. No serious AEs or deaths occurred, and all AEs resolved by study completion. The most frequently reported AE was headache, experienced by 7 participants (19%). All other AEs were reported by ≤3 participants (8%). One participant experienced multiple AEs of mild (dyspepsia, depressed level of consciousness, chest discomfort, paresthesia, dyspnea, fatigue, vomiting, and headache) and moderate (palpitations, nausea, feeling cold, dizziness, anxiety, skin burning sensation, headache, and ear pain) severity starting Day 2 after dosing in Period 1. None of the AEs were considered related to ISL, and all resolved within 11 days of onset. Nevertheless, the participant discontinued the study 52 days after ISL dosing in Period 1. There were no clinically meaningful findings in laboratory safety tests (including changes in lymphocyte counts), vital signs, or electrocardiograms during the study.

## DISCUSSION

In this Phase 1 study conducted in adults without HIV, the co-administration of multiple doses of ULO 200 mg once weekly (qw) with a single dose of ISL 2 mg did not exhibit any indication of DDIs and was generally well tolerated. Plasma ULO exposures were comparable with and without co-administration of ISL. Similarly, plasma ISL exposures remained consistent regardless of whether ISL was administered alone or with ULO. Intracellular ISL-TP concentrations in PBMCs following co-administration of ISL with ULO showed small decreases relative to ISL administered alone; however, these changes were not considered clinically meaningful. Furthermore, the mean PBMC counts following administration of ISL alone were generally lower than the mean PBMC counts following co-administration with ULO. Because ISL-TP concentrations were calculated on a per-cell basis, they were inversely correlated with PBMC counts. Therefore, the small differences observed in ISL-TP exposures may in part reflect variability in PBMC counts rather than an interaction between ULO and ISL. However, the fixed-sequence study design and variability in PBMC counts do not allow definitive differentiation between an effect attributable to PBMC counting variability and a true reduction in per-cell ISL-TP concentration. Notably, despite the modest reduction in ISL-TP exposure, C_168_ following co-administration (0.356 pmol/10^6^ cells) remained well above the established efficacy threshold of 0.05 pmol/10^6^ cells in PBMCs, indicating that the observed decrease was unlikely to be clinically meaningful (21). In addition, ULO PK was evaluated following the second weekly dose when administered alone and following the third weekly dose when co-administered with ISL. Therefore, modest increases in ULO exposure may reflect expected ULO accumulation following the additional weekly dose rather than a DDI.

Previous studies have also demonstrated a low potential for ULO to cause clinically meaningful DDIs (8, 15, 24). ULO demonstrated only weak CYP3A4 induction *in vitro,* and only a small decrease in midazolam (a substrate for CYP3A-mediated metabolism) plasma concentrations was observed after co-administration with ULO compared with midazolam administered alone (15). Together, these findings indicate that ULO has a low potential to cause clinically meaningful CYP3A-mediated DDIs.

ISL is taken up by most metabolically active cells, including PBMCs, and is phosphorylated intracellularly to its active form, ISL-TP (13). ISL is eliminated primarily via non–CYP-mediated metabolism and by renal excretion of unchanged drug (25, 26). Therefore, even if ULO was an inducer or inhibitor of CYP3A, ISL is not expected to be susceptible to CYP3A-mediated interactions. However, other drug interactions are more difficult to predict, and a clinically meaningful effect of ULO-mediated CYP3A induction on ISL exposure would not be anticipated. Accordingly, this study was designed to evaluate the overall DDI potential of ULO and ISL under clinically relevant dosing conditions rather than a specific CYP3A-mediated interaction. ISL also does not inhibit major drug-metabolizing enzymes and does not interact with drug transporters at clinically relevant concentrations (25). Similarly, multiple-dose ISL did not alter the PK of the oral contraceptive levonorgestrel/ethinyl estradiol, indicating that hormonal contraceptives and ULO can be used concomitantly (27). These findings support their potential use in combination antiretroviral therapy and co-administration with other concomitant medications (25). However, co-administration of ISL with lamivudine reduced the formation of the active metabolite, ISL-TP (28). The same effect on ISL-TP would be expected when ISL is co-administered with emtricitabine, which has a similar pharmacology to lamivudine. Thus, ISL is contraindicated when co-administered with lamivudine or emtricitabine, as significant decreases in ISL-TP concentrations may occur, which may decrease its effectiveness (28). Because plasma ISL exhibits minimal accumulation with qw dosing, close to steady-state ISL levels are reached after a single dose, and a single dose is therefore sufficient to assess ISL for DDI precipitant effects. Clinical studies have confirmed no effect of ISL on the PK of doravirine (29), dolutegravir, tenofovir disoproxil fumarate (15), atorvastatin (30), metformin (30), methadone (31), and oral contraceptives (27).

In this study, multiple oral doses of ULO co-administered with a single oral dose of ISL were generally well tolerated by adults without HIV. The combination of ISL and ULO was previously tested in a Phase 2b study (NCT04564547) in which virologically suppressed people living with HIV received a high dose of ISL (20 mg) plus ULO (100, 200, or 400 mg) qw (32). After 24 weeks of dosing, participants receiving ISL plus ULO reported similar rates of AEs, drug-related AEs, and AEs leading to study drug discontinuation as participants receiving bictegravir/emtricitabine/tenofovir alafenamide (32). However, mean decreases in total lymphocyte and CD4+ T-cell counts were observed in all groups receiving ISL 20 mg plus ULO qw, with an apparently more modest effect in the ULO 100 mg group (32). *Post hoc* modeling and simulation analyses incorporating data from across the ISL development program identified that a dose of ISL 2 mg qw is not expected to meaningfully reduce total lymphocyte or CD4+ T-cell counts (22). In Phase 2 and Phase 3 randomized studies, changes from baseline in total lymphocyte and CD4+ T-cell counts were similar between people living with HIV-1 who received ISL 2 mg plus lenacapavir 300 mg qw and those receiving daily bictegravir, emtricitabine, and tenofovir alafenamide (33, 34). In a Phase 1 study in adults without HIV, ULO 200 mg qw demonstrated an AE profile similar to that of placebo and did not decrease total lymphocyte or CD4+ T-cell counts to a clinically significant degree at Week 12 (23). An ongoing Phase 2b study (NCT06891066) is evaluating the safety of a switch to ISL 2 mg plus ULO 200 mg qw compared with continued treatment with bictegravir/emtricitabine/tenofovir alafenamide once daily, including the impact of ISL and ULO on lymphocyte counts (35).

This study has several limitations. To minimize variability, the study used intra-individual comparisons and a fixed-sequence design rather than a parallel treatment design. This approach reduced operational complexity, incorporating multiple doses of ULO to achieve any possible enzyme induction, but introduced the potential for a period effect; that is, differences in study outcomes could be attributed to the timing of drug administration or assessments rather than the pharmacologic properties of the drug itself. Indeed, the variability in PBMC counts from Period 1 to Period 3 likely contributed to the modest difference observed in PBMC ISL-TP PK. A crossover design might have overcome this potential limitation but was not implemented due to the complexities associated with the long half-lives of both ISL and ULO, including coordination of multiple dosing schedules and ensuring adequate washout periods. Furthermore, as discussed above, only a single dose of ISL was implemented in the co-administration Period 3 to assess the DDI precipitant potential of ISL. The steady-state PK of ISL-TP in PBMCs was not measured because this would have been impractical given the prolonged ISL-TP half-life (195 h) (17). In addition, intracellular ISL-TP is not mechanistically expected to affect ULO exposure because it is confined within hematopoietic cells, such as PBMCs, which have the necessary phosphorylation enzymes to generate ISL-TP. It is therefore unlikely that ISL-TP and ULO would be able to interact with drug-metabolizing enzymes or transporters involved in ULO disposition (17). However, the PK of ISL and ISL-TP following single- and multiple-dose administration has been well characterized and may be used to project the PK profile at administration (7, 17). The observed ISL-TP half-life (156–176 h) was shorter than the previously reported 195 h, which is likely attributable to normal variability in terminal half-life estimation due to differences in handling of PBMCs and the duration of PK sampling (17).

Finally, as this study was conducted in adults without HIV-1, caution should be exercised when extrapolating these findings to people living with HIV-1 whose lymphocyte counts and PBMC composition may differ from those of adults without HIV.

### Conclusions

Co-administration of ULO with ISL did not meaningfully affect the PK profiles of either drug. Multiple doses of ULO 200 mg qw co-administered with a single dose of ISL 2 mg were generally well tolerated in adult participants without HIV. The results of this study support the continued clinical development of ULO 200 mg in combination with ISL 2 mg oral qw for the treatment of HIV-1.

## MATERIALS AND METHODS

### Study design

This open-label, three-period, fixed-sequence, two-way Phase 1 study in adults without HIV (protocol MK-8507-016, NCT06619678; Fig. 1) was conducted between January and June 2024. The study was conducted in accordance with the principles of Good Clinical Practice and was approved by the appropriate institutional review boards and regulatory agencies. Written informed consent was obtained before participant screening.

Male and non-pregnant, non-breastfeeding female adults 19–65 years old, with a body mass index (BMI) of ≤32 kg/m^2^ at screening were eligible for enrollment. Key exclusion criteria were history of clinically significant abnormalities or diseases; significant multiple and/or severe allergies; an inability to refrain from the use of any medication; any recent major surgeries; and positive test results for hepatitis B surface antigen, hepatitis C antibodies, or HIV antibodies. Screening of participants occurred within 28 days prior to the first dose in Period 1. The complete list of inclusion and exclusion criteria is provided in the Supplemental Materials S1.

In Period 1, eligible participants received a single dose of ISL 2 mg on the morning of Day 1. In Period 2, approximately 6 weeks after receiving ISL in Period 1, participants received ULO 200 mg on the morning of Day 1 and again on Day 8. In Period 3, a single dose of ISL 2 mg was co-administered with ULO 200 mg 7 days after the Day 8 dose of ULO in Period 2, followed by dosing of ULO 200 mg qw on Days 8, 15, 22, and 29 (without any additional doses of ISL). In Periods 1 and 2, and Day 1 of Period 3, all doses of ISL and ULO were administered at the study site following an 8-h fast. All doses of ISL and ULO were administered orally with approximately 240 mL of water at the same time each day. The total planned study duration from screening to the end of follow-up for each participant was 18 weeks (Fig. 1).

The sample size for plasma ULO was based on assumed within-participant standard deviation (SDs) of 0.067 for C_168_ and 0.035 for AUC_0–168_, based on data from MK-8507-008 (8). The sample size for PBMC ISL-TPs was based on assumed within-participant SDs of 0.362 for C_168_ and 0.289 for AUC_0–168_, based on data from MK-8591-002 (29). Approximately 30 participants completing the study were expected to provide sufficiently precise estimates of the GMRs and corresponding 90% *CIs* for ULO administered with or without ISL, and for ISL administered with ULO versus alone.

### Study objectives and endpoints

The primary objectives were (1) to evaluate the PK of ULO in plasma following administration of multiple doses of ULO qw alone or with a single dose of ISL, and (2) to evaluate the PK of ISL in plasma and ISL-TP in PBMCs following administration of ISL alone or with multiple doses of ULO qw. The primary endpoints for ULO in plasma were AUC_0-168_, C_max_, C_168_, T_max_, t_1/2_, apparent clearance after dosing (CL/F), and apparent volume of distribution during the terminal phase after dosing (Vd/F). The primary endpoints for ISL in plasma and ISL-TP in PBMCs were AUC_0–∞_, AUC_0–168_, C_max_, C_168_, T_max_, and t_1/2_; ISL in plasma also included CL/F and Vd/F as the primary endpoints. For AUC_0–∞_, AUC_0–168_, C_max_, and C_168_, the GMR was calculated for co-administration versus ISL or ULO alone, along with the associated two-sided 90% *CI*. In a *post hoc* analysis, the 90% *CIs* for the GMRs were compared to the standard bioequivalence bounds of 0.8 to 1.25 (36).

The secondary objective was to assess the safety and tolerability of the co-administration of ULO and ISL in adults without HIV. Safety was monitored throughout the study via clinical and laboratory evaluations. The secondary endpoints were the incidence of AEs and discontinuations due to AEs.

PK analyses were based on plasma samples from all participants who adhered to the study protocol up to the relevant sampling period. Safety and tolerability analyses included all participants who received at least one dose of study intervention.

### Assessments

In Period 1, serial blood samples were collected on Day 1 for measurement of plasma ISL concentrations (predose and at 0.25, 0.5, 1, 2, 4, 8, 12, 24, 48, 96, 120, 144, 168, 336, and 504 h after dose), and for measurement of PBMC ISL-TP concentration (predose and at 4, 8, 12, 24, 96, 120, 144, 168, 336, 504, 672, 840, and 1,008 h after dose). In Period 2, blood samples for measurement of plasma ULO concentrations were collected on Day 1 (predose) and on Day 8 (predose and at 1, 2, 4, 6, 8, 12, 24, 48, 96, 120, and 168 h after the Day 8 dose). In Period 3, blood samples were collected for measurement of plasma ULO concentrations (predose and at 1, 2, 4, 6, 8, 12, 24, 48, 96, 120, 168, 336, 504, 672, 840, and 1,008 h after the Day 1 dose), and for measurement of plasma ISL and PBMC ISL-TP concentrations at the same times as described for Period 1 above.

Sample collection, handling, labeling, storage, and shipment apply to both the ISL and ULO assays. Blood samples were collected in labeled 4 mL blood collection tubes containing K_2_ ethylenediaminetetraacetic acid as the anticoagulant. The blood-containing tubes were gently inverted 8–10 times and placed immediately in an ice water bath. The samples were maintained in an ice bath throughout sample collection and until further processing. Within 30 min of collection, the blood samples were centrifuged at 1000–1300 × g for approximately 10 min and at 4°C (range, 2°C to 8°C). Samples that were interrupted during centrifugation, disturbed during the separation process, or exhibited inadequate separation were re-spun under the same conditions to separate the maximum amount of plasma from each sample. Immediately after separation of the whole blood, the plasma was carefully transferred into two labeled cryotubes identified with pre-printed barcoded labels dividing plasma equally. The samples were maintained in an ice bath following centrifugation and until storage in the freezer at −20°C (±15°C) until transfer to Syneos. The storage of aliquots was completed within 60 min from the time of the blood draw.

Safety and tolerability were evaluated based on AE monitoring, including discontinuations due to AEs, physical examinations, vital signs, electrocardiography, and laboratory safety tests conducted or collected throughout the study. Vital signs and 12-lead electrocardiogram results were assessed during screening; predose; at Day 15 of Period 1; at Days 1 and 8 of Period 2; and at Days 1, 15, and 43 of Period 3. Hematology (including lymphocyte counts), urinalysis, and chemistry were monitored at screening; predose; at Day 15 of Period 1; at Days −1 and 7 of Period 2; and at Days −1 and 15 of Period 3. All clinical AEs reported by the participant or observed by the investigator were graded as mild, moderate, or severe in intensity and were assessed by the investigator for relationship to study drug.

### PK analysis

ISL and ULO concentrations in plasma and ISL-TP concentrations in PBMCs were determined using high-performance liquid chromatography-tandem mass spectrometry methods that were validated for accuracy, precision, linearity, sensitivity, and specificity by Syneos Health Clinique Inc. (Québec City, Québec, Canada). The analytical range was 0.02 to 20 ng/mL (0.0682–68.2 nM) for ISL, 1 to 1000 ng/mL for ULO (2.03–2030 nM), and 0.1 to 40 ng/mL (0.188–75.0 nM) for ISL-TP. Values below the limit of quantification for each drug were treated as zero. If a value was an obvious outlier, it was treated as missing. AUC_0–∞_ and t_½_ were set to missing because the elimination phase was not well characterized (e.g., adjusted r^2^ <0.75, fluctuations of concentrations at the end of the terminal phase). PBMC cell counts were determined by Celerion (Lincoln, Nebraska, USA). PBMC ISL-TP concentrations (nM) were corrected for cell count (pmol/10^6^ cells) prior to performing PK analysis.

### Statistical analysis

PK parameter values were calculated by noncompartmental analyses using Phoenix WinNonlin software, version 8.4 (Certara USA, Inc., Princeton, NJ, USA). C_max_, C_168_, and T_max_ values were obtained directly from the plasma concentration-time data. AUCs were calculated using the linear-up, log-down method. The t_1/2_ was calculated as ln (2)/λz, where λz is the apparent first-order terminal elimination rate constant calculated from the slope of the linear regression of the terminal log-linear portion of the plasma concentration-time profile. For each of the following parameters, individual values were natural log–transformed and assessed with a linear mixed-effects model containing a fixed effect for treatment and a random effect for participants: plasma ULO C_168_, AUC_0–168_, and C_max_, plasma ISL AUC_0–∞_, AUC_0–168_, and C_max_, and PBMC ISL-TP AUC_0–∞,_ AUC_0–168_, and C_max_. An unstructured covariance matrix was used to account for association within repeated measurements. The Kenward-Roger adjustment was used to calculate the denominator degrees of freedom for the fixed effect.

## Data Availability

The data sharing policy, including procedures and restrictions, of Merck Sharp & Dohme LLC, a subsidiary of Merck & Co., Inc., Rahway, NJ, USA (MSD), is available at https://externaldatasharing-msd.com/.

## SUPPLEMENTAL MATERIAL

Supplemental material aac.00676-26-s0001.docx Table S1.

ASM does not own the copyrights to Supplemental Material that may be linked to, or accessed through, an article. The authors have granted ASM a non-exclusive, world-wide license to publish the Supplemental Material files. Please contact the corresponding author directly for reuse.
