# Supplementary material for: Open-label Phase 1 study to evaluate drug-drug interactions between ulonivirine and islatravir in adults without HIV
Source: Antimicrob Agents Chemother. 2026 Oct 8;70(11):e00676-26. doi: 10.1128/aac.00676-26 (PMC13731176; doi:10.1128/aac.00676-26)
Supplement: Supplementary file 1 [file aac.00676-26-s0001.docx]

**Supplemental Materials**

**Eligibility criteria**

**Inclusion criteria**

An individual was eligible for inclusion in the study if the individual met all of the following

criteria:

1. The participant was in good health based on medical history, physical examination, vital sign measurements, and electrocardiograms performed before randomization.
2. The participant was in good health based on laboratory safety tests obtained at the screening visit and before administration of the initial dose of study intervention.
3. Body mass index ≤32 kg/m^2^.
4. The participant was an individual of any sex/gender, from 19 to 65 years of age inclusive, at the time of providing the informed consent.
5. A participant assigned female sex at birth was eligible to participate if not pregnant or breastfeeding, and at least 1 of the following conditions applied:

- Was not a woman of childbearing potential (WOCBP)
- Was a WOCBP and used an acceptable contraceptive method or abstained from penile-vaginal intercourse as their preferred and usual lifestyle (abstinent on a long-term and persistent basis) during the intervention period and for at least 14 days after the last dose of study intervention. Had a negative highly sensitive pregnancy test (urine or serum) as required by local regulations within 24 h (for a urine test) or 72 h (for a serum test) before the first dose of study intervention

6. The participant provided documented informed consent for the study, including for future biomedical research.

**Exclusion criteria**

An individual was excluded from the study if the individual met any of the following criteria:

1. The participant had a history of clinically significant endocrine, gastrointestinal, cardiovascular, hematologic, hepatic, immunologic, renal, respiratory, genitourinary, or major neurologic (including stroke and chronic seizures) abnormalities or diseases. Participants with a remote history of uncomplicated medical events (eg, uncomplicated kidney stones, as defined as spontaneous passage and no recurrence in the last 5 years, or childhood asthma) were enrolled in the study at the discretion of the investigator.
2. The participant was mentally or legally incapacitated, had significant emotional problems at the time of screening visit or expected during the conduct of the study, or had a history of clinically significant psychiatric disorder of the last 5 years. Participants who have had situational depression were enrolled in the study at the discretion of the investigator.
3. The participant had a history of cancer (malignancy). Participants with adequately treated disease deemed as “cured” or who, in the opinion of the study investigator, were highly unlikely to sustain a recurrence for the duration of the study were enrolled at the discretion of the investigator.
4. The participant had a history of significant multiple and/or severe allergies (eg, food, drug, latex allergy) or had an anaphylactic reaction or significant intolerability (i.e., systemic allergic reaction) to prescription or nonprescription drugs or food.
5. The participant had positive test(s) results for hepatitis B surface antigen, hepatitis C antibodies, or HIV.
6. The participant had a major surgery and/or donated or lost 1 unit of blood (approximately 500 mL) within 4 weeks prior to the screening visit.
7. The participant was unable to refrain from or anticipated the use of any medication, including prescription and nonprescription drugs or herbal remedies beginning approximately 2 weeks (or 5 half-lives) prior to administration of the initial dose of study intervention, throughout the study (including washout intervals between treatment periods), until the poststudy visit.
8. The participant participated in another investigational study within 4 weeks (or 5 half‑lives, whichever was greater) prior to the screening visit. The window was derived from the date of the last visit in the previous study.
9. The participant had a corrected QT interval ≥470 msec (for males) or ≥480 msec (for females).
10. The participant was under the age of legal consent.
11. The participant was a smoker and/or had used nicotine or nicotine-containing products (e.g., nicotine patch and electronic cigarette) within 3 months of screening.
12. The participant consumed greater than 3 servings of alcoholic beverages (1 serving was approximately equivalent to: beer [354 mL/12 ounces], wine [118 mL/4 ounces], or distilled spirits [29.5 mL/1 ounce]) per day. Participants who consumed 4 servings of alcoholic beverages per day were enrolled at the discretion of the investigator.
13. The participant consumed excessive amounts, defined as greater than 6 servings (1 serving is approximately equivalent to 120 mg of caffeine) of coffee, tea, cola, energy drinks, or other caffeinated beverages per day.
14. The participant was a regular user of cannabis or any illicit drugs or had a history of drug (including alcohol) abuse within approximately 2 years. Participants had a negative drug screen prior to randomization.
15. The investigator had any concern regarding safe participation of the participant in the study or had any other reason of concern where the investigator considered the participant inappropriate for participation in the study.
16. Was or had an immediate family member (eg, spouse, parent/legal guardian, sibling, or child) who was investigational site or Sponsor staff and directly involved with this study.

**Table S1.** **Summary of PBMC counts following a single oral dose of ISL alone and following co-administration with multiple doses of ULO qw**

|  | **ISL** | | | **ISL+ULO** | | |
| --- | --- | --- | --- | --- | --- | --- |
| **Sample times** | ***n*** | **GM,**  **10^6^ cells** | **GCV,**  **%** | ***n*** | **GM,**  **10^6^ cells** | **GCV,**  **%** |
| Predose Day 1 | 36 | 7.0 | 87.3 | 35 | 3.6 | 469.2 |
| 4 h | 36 | 6.5 | 81.3 | 35 | 9.4 | 61.0 |
| 8 h | 36 | 14.3 | 67.6 | 35 | 11.1 | 98.4 |
| 12 h | 36 | 10.7 | 68.8 | 35 | 15.8 | 71.5 |
| 24 h | 36 | 5.4 | 103.9 | 35 | 5.1 | 87.0 |
| 96 h | 36 | 4.7 | 86.8 | 35 | 9.9 | 88.0 |
| 120 h | 36 | 6.4 | 95.2 | 35 | 6.6 | 85.4 |
| 144 h | 36 | 5.2 | 104.9 | 35 | 6.5 | 81.5 |
| 168 h | 36 | 6.5 | 84.3 | 35 | 9.2 | 67.4 |
| 336 h | 36 | 5.4 | 158.3 | 34 | 8.9 | 99.9 |
| 504 h | 35 | 6.2 | 116.3 | 35 | 7.2 | 105.2 |
| 672 h | 35 | 9.3 | 105.7 | 35 | 7.7 | 89.3 |
| 840 h | 35 | 7.2 | 84.5 | 35 | 9.4 | 73.1 |
| 1008 h | 35 | 8.3 | 71.3 | 35 | 8.4 | 86.4 |
| 0 to 1008 h (all time points) | 36 | 8.5 | 51.3 | 35 | 10.1 | 41.1 |
| 4 to 1008 h (all time points) | 36 | 8.6 | 50.5 | 35 | 10.4 | 39.8 |

GCV, geometric coefficient of variation; GM, geometric mean; ISL, islatravir; PBMC, peripheral blood mononuclear cell; qw, once weekly; ULO, ulonivirine.
